# A rare case of viral-associated trichodysplasia spinulosa in a patient with chronic lymphocytic leukemia

**DOI:** 10.1097/JW9.0000000000000069

**Published:** 2023-02-23

**Authors:** Rachel Choi, Shaman Bhullar, Jennifer McNiff, Justin Persico, Jonathan Leventhal

**Affiliations:** a Department of Dermatology, Yale University School of Medicine, New Haven, Connecticut; b Department of Pathology, Yale University School of Medicine, New Haven, Connecticut; c Department of Medical Oncology, Yale University School of Medicine, New Haven, Connecticut

**Keywords:** trichodysplasia spinulosa, trichodysplasia spinulosa-associated polyomavirus, chronic lymphocytic leukemia, immunodeficiency, cidofovir

## Dear Editors,

Viral-associated trichodysplasia spinulosa (VATS) is a cutaneous eruption of folliculo-centric shiny papules and alopecia that most commonly occurs in transplant recipient patients.^1^ Here, we present an extremely rare case of VATS in a female patient with chronic lymphocytic leukemia (CLL). An informed consent was obtained from the patient for this report.

**Fig. 1.**
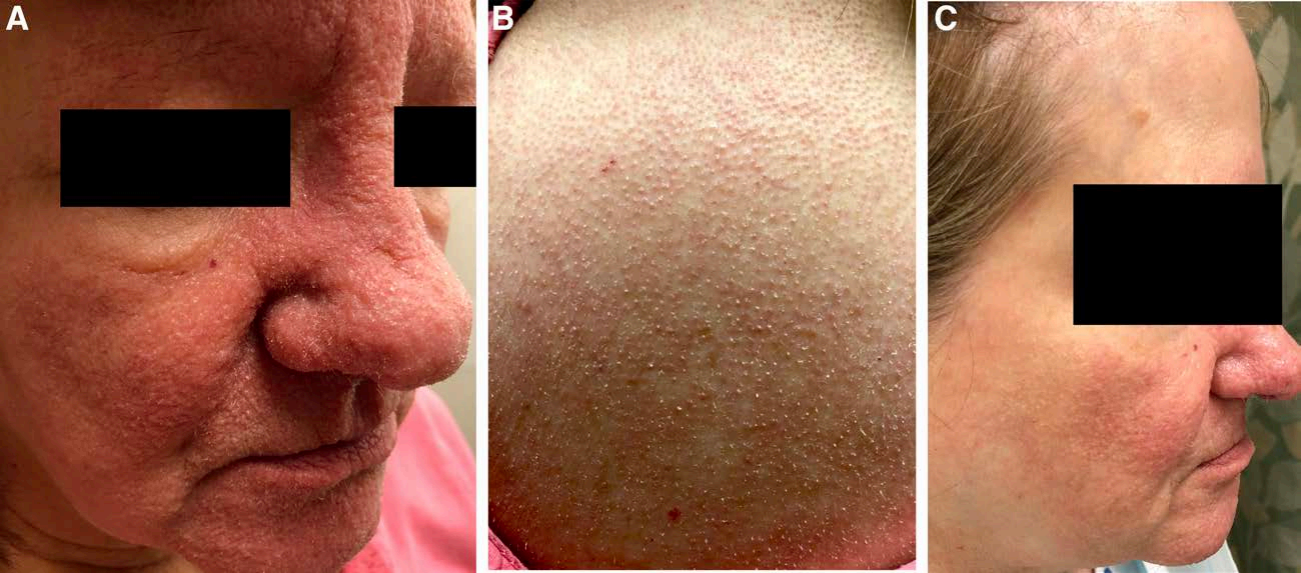
Clinical progression of viral-associated trichodysplasia spinulosa treated with topical cidofovir. Spiny red papules and spicules concentrated on face (A) and upper back (B) found on initial presentation. Face showing dramatically decreased spicules and papules, particularly on cheek and forehead, after 9 months of treatment with topical cidofovir (C).

**Fig. 2.**
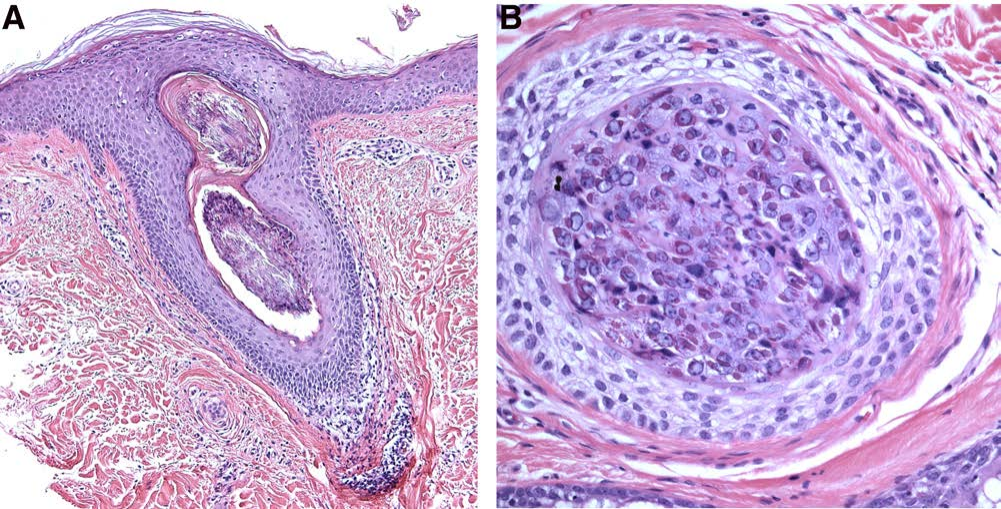
Pathological findings of viral-associated trichodysplasia spinulosa showing unusual prominent trichohyalin granules with disruption of the normal follicular maturation showing basophilic inclusions within the follicular ostia, shown at magnification 100× (A) and 400× (B).

A 63-year-old female presented with a 2-month history of multiple reddish-pinkish papules on her face in the setting of long-standing CLL. Physical examination also showed leonine facies, superciliary madarosis, and an infiltrative nose. Many spiny red papules and spicules were located on her face, neck, chest, arms, and back (Fig. 1A and B). A punch biopsy of one of the lesions on the back revealed prominent trichohyalin granules with disruption of the normal follicular maturation showing basophilic inclusions within the follicular ostia consistent with VATS (Fig. 2A and B).

Importantly, the patient was not on any immunosuppressive therapy when she developed the VATS lesions. The most recent treatment for her CLL had been approximately 1 year ago, when she had finished 6 cycles of bendamustine plus rituximab. Prior to that round of treatment, her small lymphocytic lymphoma had been diagnosed over 20 years ago but had not required therapy for 10 years.

A complete work up for immunosuppression including HIV testing, T-cell subsets, immunoglobulins, and re-staging of small lymphocytic lymphoma was performed. She was negative for HIV-1/2 antibody/HIV-1 antigen screening. She had evidence of hypogammaglobulinemia with an IgG of 667 mg/dL and low IgG subclasses 2, 3, and 4 (72, 16, and 1.3 mg/dL, respectively). IgG subclass 1 was normal (482 mg/dL). A peripheral blood flow cytometry showed absolute T-cell lymphopenia with low CD3+ and CD3+CD4+ absolute counts (504 and 267.2/µL, respectively), but no evidence of lymphoproliferative disease. Laboratory values showed a low absolute lymphocyte count of 0.8/µL.

Prior treatment of the skin lesions by the primary care physician included topical corticosteroids, topical metronidazole, and oral cephalexin, and were not effective. After the diagnosis was established, the patient was treated with 450 mg valganciclovir twice daily to target the underlying polyomavirus, but showed no improvement over 4 months. 3% topical cidofovir twice daily was then added, and the patient showed significant improvement over the following 9 months on both 450 mg valganciclovir twice daily and 3% topical cidofovir treatment (Fig. 1C). During this period, valganciclovir was discontinued by the patient for a period of 3 months due to apparent lack of efficacy but was restarted based on dermatology recommendation. The plan of care is to continue this treatment indefinitely as maintenance therapy.

Although VATS has been reported frequently in transplant recipient patients and patients with acute lymphocytic leukemia and non-Hodgkin’s lymphoma, there are only 3 previously reported account of VATS in a patient with CLL in the literature.^2–4^ Importantly, the previously reported patient developed VATS just 2 months after ceasing fludarabine,^3^ whereas our patient had been off immunosuppressive agents for nearly 1 year prior to developing VATS. Thus, our unique case highlights the dermatologically significant impact of CLL-induced immunodeficiency. The etiology of the immunodeficiency caused by CLL is multifactorial, including hypogammaglobulinemia, hypocomplementemia, and altered major histocompatibility complex class II antigens on cancer cells.^5^ Finally, this case emphasizes the important role that topical cidofovir may have in treating patients with treatment-resistant VATS.^6^

## Conflicts of interest

None.

## Funding

None.

## Study approval

N/A.

## Author contributions

RC and SB: Writing and editing manuscript. JM and JP: Interpretation of information and editing. JL: Editing manuscript, supervision.
